# The Emerging Role of the Microenvironment in Endometrial Cancer

**DOI:** 10.3390/cancers10110408

**Published:** 2018-10-30

**Authors:** Subhransu S. Sahoo, Xu Dong Zhang, Hubert Hondermarck, Pradeep S. Tanwar

**Affiliations:** 1 Gynecology Oncology Group, University of Newcastle, Callaghan, NSW 2308, Australia; subhransu.sahoo@uon.edu.au; 2 School of Biomedical Sciences and Pharmacy, University of Newcastle, Callaghan, NSW 2308, Australia; Xu.Zhang@newcastle.edu.au (X.D.Z.); hubert.hondermarck@newcastle.edu.au (H.H.)

**Keywords:** endometrial cancer, TME, stromal cells, metastasis, chemoprevention

## Abstract

Endometrial cancer (EC) is one of the most frequently diagnosed cancers in women, and despite recent therapeutic advances, in many cases, treatment failure results in cancer recurrence, metastasis, and death. Current research demonstrates that the interactive crosstalk between two discrete cell types (tumor and stroma) promotes tumor growth and investigations have uncovered the dual role of the stromal cells in the normal and cancerous state. In contrast to tumor cells, stromal cells within the tumor microenvironment (TME) are genetically stable. However, tumor cells modify adjacent stromal cells in the TME. The alteration in signaling cascades of TME from anti-tumorigenic to pro-tumorigenic enhances metastatic potential and/or confers therapeutic resistance. Therefore, the TME is a fertile ground for the development of novel therapies. Furthermore, disrupting cancer-promoting signals from the TME or re-educating stromal cells may be an effective strategy to impair metastatic progression. Here, we review the paradoxical role of different non-neoplastic stromal cells during specific stages of EC progression. We also suggest that the inhibition of microenvironment-derived signals may suppress metastatic EC progression and offer novel potential therapeutic interventions.

## 1. Introduction

Worldwide, endometrial cancer (EC) is the most prevalent invasive gynecologic malignancy [1]. Although early diagnosis, surgery, and chemotherapy have reduced EC mortality, still, in many cases, these patients eventually succumb to their malignancy. The mechanisms involved with the aggressive transformation of tumor cells are poorly understood. The molecular signals derived from stromal cells and/or the extracellular matrix (ECM) play an important role in the progression of an indolent tumor to a malignant state [2,3]. The interaction between tumor cells and the tumor microenvironment (TME) regulates cancer progression of almost all types of cancer [4,5]. This concept was proposed early, in 1889 by Stephen Paget in his “seed and soil” hypothesis, which suggests that a seed (tumor cells) can only grow in a fertile soil (microenvironment) [6]. Similarly, tumor cells (seed) can thrive only where microenvironment (soil) is somewhat favorable.

Dynamic reciprocity between cells and their microenvironment is crucial for both normal tissue homeostasis and tumor growth [7]. The tissue microenvironment consists of both cellular (fibroblasts, myofibroblasts, blood vessels, pericytes, adipocytes, smooth muscle cells, immune and inflammatory cells) and non-cellular (ECM) components [8]. In the normal state, cells exchange information with other cell types by direct cell-cell contact or through ECM [9]. The ECM is a repository of growth factors, cytokines, and structural proteins, which are produced as a consequence of the crosstalk between the epithelial cells and the surrounding stromal cells [10]. The basal surface of epithelial cells forms the basement membrane, which separates the epithelial and stromal compartments. In this manner, a normal well-differentiated epithelium is separated by a well-delineated basement membrane from the dermal or stromal compartment [11]. However, during the transition to a pre-malignant state or progression to carcinoma, the normal tissue homeostasis gets disturbed, which results in proliferation of epithelial cells and invasion of these tumor cells to the stromal compartment through the degraded basement membrane [11]. Furthermore, the crosstalk of soluble factors or proteins between tumor cells and non-cancerous stromal cells supports tumor development and progression [12,13]. Consequently, tumor cells reorient the surrounding stroma to change it from a restrictive to a supportive TME, eventually promoting the dissemination of tumor cells.

**Figure 1:**
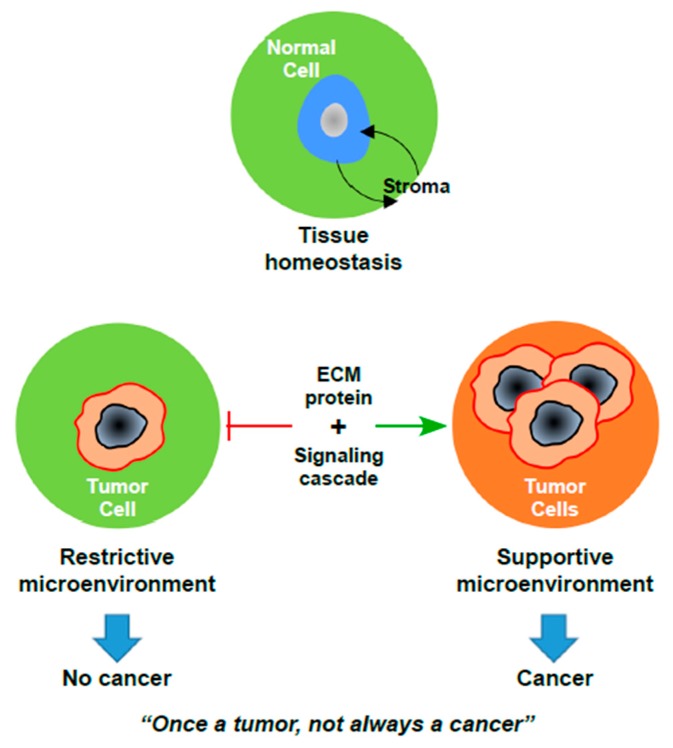
The paradox of cancer development. Upon loss of tissue homeostasis, the progression of an occult tumor to frank carcinoma requires significant changes in the microenvironment. ECM: Extracellular Matrix.

Thus, the tissue microenvironment has reciprocal functions in the healthy and diseased states. In a healthy state, the normal microenvironment provides antitumorigenic signals to maintain epithelial tissue homeostasis [14]. However, during the progression of cancer, the reactive stromal components promote tumor cell proliferation through diverse signaling cascades. Moreover, oncogenic mutations in tumor cells are not sufficient to drive a high-grade cancerous state unless the molecular signaling cascades have been perturbed by the microenvironment, see Figure 1. Thus, the TME has a significant contribution towards driving tumor progression. In this review, we discuss the current understanding of endometrial carcinoma from a microenvironment vantage point, highlighting the stromal cell-derived signaling cascades involved in the progression of high-grade EC.

## 2. Endometrial Cancer Microenvironment

Carcinogenesis is a multistep process, starting from the initial carcinogenic stimulus to the final manifestation of cancer. Although uncontrolled growth is a fundamental characteristic of cancer cells, these cells also require a proper microenvironment to survive and develop [14]. Like in most cancers, the TME also contributes a pivotal role in EC progression [15]. Indeed, mutations in *PTEN*, *KRAS*, *p53*, and microsatellite instability initiates EC lesions, but this does not lead to high-grade cancer or metastasis unless supported by the microenvironment [16]. The microenvironment of EC cells is populated by diverse cell types including fibroblasts, myofibroblasts, endothelial cells, macrophages, and inflammatory cells, see Table 1 [15]. These cells communicate with EC cells through cytokines, growth factors, or receptors for ligand binding secreted from EC cells. Thus, the reciprocal interactions between EC cells and various stromal cells generate a favorable microenvironment conducive to invasion and metastasis. Invasion and metastasis of tumor cells are one of the main reasons for treatment failure and poor prognosis in EC patients. The identification of microenvironment-derived signals or stromal cell-derived proteins can potentially serve as biomarkers for high-grade metastatic EC. In this review, we address the role of various stromal proteins and pathways which contribute to endometrial carcinogenesis. 

### 2.1. The Role of Stromal Myofibroblasts in EC Microenvironment

**Figure 2:**
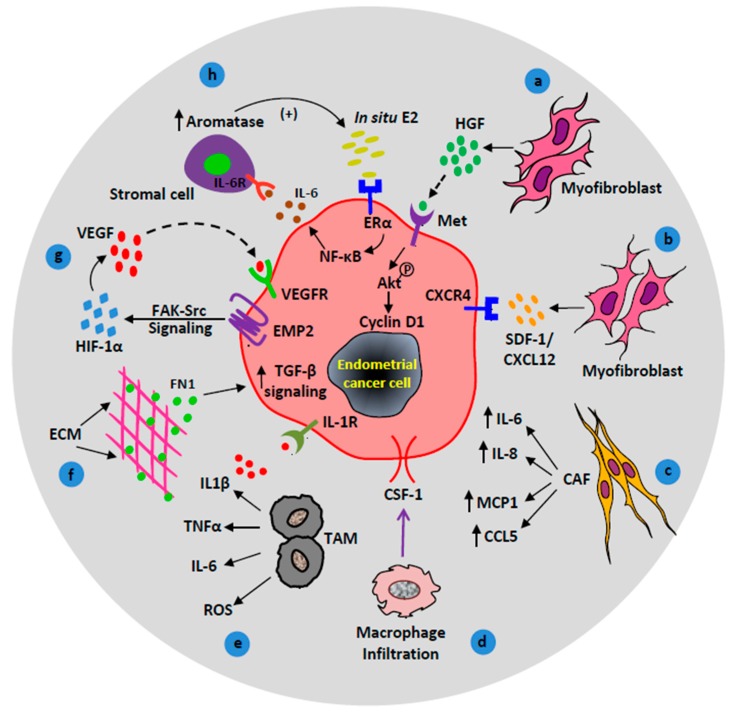
Contributions of activated/recruited stromal cells in endometrial cancer (EC) progression. (**a**) Hepatocyte growth factor (HGF) stimulates proliferation of EC cells via the HGF/c-MET/AKT signaling pathway. (**b**) Myofibroblasts promote tumor growth via CXCR4/CXCL12 signaling axis. (**c**) Cancer-associated fibroblasts (CAF) secrete cytokines (IL-6, IL-8, and MCP-1) and chemokines (CCL5/RANTES) to promote cancer progression. (**d**) Macrophage response, colony-stimulating factor 1 (CSF1) signals macrophage infiltration to the endometrial reactive stroma. (**e**) Tumor-associated macrophages contribute to endometrial carcinogenesis via the production of cytokines (IL1β, TNFα, IL-6) and reactive oxygen species (ROS). (**f**) Extracellular matrix (ECM) protein, fibronectin upregulates transforming growth factor-beta (TGF-β) signaling in EC cells which facilitates EC metastasis. (**g**) Under hypoxic conditions, epithelial membrane protein-2 (EMP2) enhances angiogenesis through focal adhesion kinase (FAK)-Src and hypoxia-inducible factor 1-alpha (HIF-1α) signaling pathway. (**h**) A positive feedback loop between in situ estrogen (E_2_), IL-6, and aromatase upregulate EC cell proliferation.

Out of several other stromal cells, myofibroblasts have a dominant contribution in cancer progression [17,18]. Stromal myofibroblasts secrete a diverse milieu of cytokines and growth factors to boost EC growth, motility, angiogenesis, and metastasis. Hepatocyte growth factor (HGF), in particular, is secreted by myofibroblasts and is a potent growth-promoter that plays an important role in the microenvironment of EC [19,20]. Studies have demonstrated the interaction between endometrial stromal cells and EC cells through the HGF/MET pathway [21]. Endometrial myofibroblasts secrete HGF, which interacts with its receptor MET on EC cells to induce the invasion of EC cells [21,22]. Furthermore, in a recent study, both ex vivo and in vivo experiments show the activation of the HGF/c-MET/AKT signaling pathway in EC [22]. Phosphorylation of the Met receptor by HGF further phosphorylates downstream AKT protein, which promotes the proliferation of epithelial cells via the modulation of cyclin D1 transcription [22], see Figure 2a. This also explains why in vitro assays that show highly significant results with AKT/PI3K inhibitors in endometrial epithelial cell cultures have failed to translate into the clinic, because stromal inputs are missing in in vitro conditions.

Furthermore, increasing evidence suggests that myofibroblasts stimulate tumor progression through CXCL12 secretion [23]. The chemokine CXCL12 (also known as stromal-derived factor-1, SDF-1) plays a critical role of chemoattractant in the tumor niche. It primarily binds to its cognate receptor CXCR4 to regulate trafficking of both normal and malignant cells. Thus, in a paracrine manner, CXCL12 attracts CXCR4 expressing tumor cells to a new tumor niche resulting in the invasion and metastasis of tumor cells, see Figure 2b [24,25]. In addition, immunohistochemistry and real-time quantitative PCR studies have also shown an elevated level of CXCR4 mRNA in human EC patient tissue samples [24,25]. These data suggest that interaction between CXCL12 and CXCR4 on an endometrial tumor cell triggers tumor cell invasion.

Several studies have also demonstrated the significant contribution of cancer-associated fibroblasts (CAF) in EC. Tumor-derived growth factors such as transforming growth factor-beta (TGF-β) differentiates stromal fibroblasts into myofibroblasts. Myofibroblasts acquire a higher level of the alpha smooth muscle actin (αSMA) protein and turn into cancer-associated fibroblasts (CAF) [5,26]. In the case of EC, the number of CAF increases with pro-malignant features. CAF in active stroma secretes higher levels of collagen I and III than those of the normal tissue, which facilitates desmoplasia by deposition of a dense collagen matrix. In addition, CAF contributes significantly to the progression of EC by chronic secretion of cytokines such as IL-6, IL-8, monocyte chemotactic protein-1 (MCP-1 or CCL2), chemokine ligand 5 (CCL5 or RANTES), and vascular endothelial growth factor (VEGF) [27,28], see Figure 2c. VEGF is a potent growth factor that stimulates vasculature around the tumor and supports angiogenesis. The secreted cytokines also act as a chemoattractant for the migration and invasion of EC cells from primary sites to secondary sites. 

In summary, stromal myofibroblasts and CAF enhance EC growth and metastasis, which suggests a significant contribution of the microenvironment in EC progression.

### 2.2. Macrophages in EC Microenvironment

Macrophages are one of the major stromal components, and they release several growth factors, cytokines, and chemokines, which facilitates tumor growth and invasion. Depending upon phenotypic diversity, macrophages have a dual role in cancer and can either promote or inhibit cancer progression. Typically, macrophages exist in two basic phenotypes, M1 macrophages with their cytotoxic potential are considered as anti-tumor phenotype and M2 macrophages associated with wound healing and tissue repair function are regarded as the pro-tumor phenotype [29]. A growing body of evidence suggests the vital role of tumor-associated macrophages (TAM) in neoplastic transformation and progression of EC [30,31,32,33]. Endometrial carcinomas have a higher macrophage density than benign endometrium [30]. Comparatively, high-grade endometrioid carcinomas or type II EC with myometrial invasion have more stromal M2 TAMs than type I endometrioid adenocarcinomas without myometrial invasion [31]. EC cell-derived chemoattractants, such as colony stimulating factor-1 (CSF-1) and the CC chemokines help in the oncogenic recruitment of the macrophages through blood vessels [44], see Figure 2d. Moreover, immunohistochemistry and tissue microarray studies have shown the presence of three macrophage response markers (CD163, FCGR2A, and FGCR3A) in endometrioid EC cells [44]. Investigations have also demonstrated that expression of CSF-1 on EC cells facilitates infiltration of mononuclear macrophages. In addition to the recruited macrophages, in situ macrophages in the uterus significantly contribute to EC progression. Macrophages reside in the peri-necrotic and perivascular areas of the uterus and promote endometrial carcinogenesis by the production of pro-inflammatory cytokines such as tumor necrosis factor-α (TNF-α), interleukin 1 beta (IL-1β), interleukin 6 (IL-6), and oxygen free radicals [45]. IL-1β signals through the IL-1β receptor (IL-1R) on the EC cell surface, see Figure 2e. The accumulation of TAMs in necrotic regions is characterized by low oxygen tension or hypoxic TME which further drives angiogenesis [45]. Thus, TAMs have a potential contribution to endometrial carcinogenesis via the production of cytokines, reactive oxygen species, and the establishment of a hypoxic microenvironment, which altogether triggers the process of angiogenesis [32]. Therefore, in addition to uncontrolled tumor cell division, macrophage-derived cytokines promote tumor cell growth and spread to secondary sites.

### 2.3. Stromal Signaling in EC Microenvironment

#### 2.3.1. ECM-Derived TGF-β Signaling

Other than cellular components of the microenvironment, non-cellular components such as ECM play an important role in fibrosis and EC metastasis. Our recent investigations show that ECM-derived TGF-β signaling promotes EC metastasis [46]. Out of several ECM proteins, fibronectin (FN1) activates the TGF-β pathway in EC cells, see Figure 2f. Our study also highlights a surplus deposition of fibronectin protein at metastatic sites of human EC patients compared to the primary origin of the tumor (uterus), and that suppression of the TGF-β pathway significantly impairs EC cell invasion and metastasis [46]. Thus, inhibition of microenvironment-derived signals can reduce EC metastasis. 

#### 2.3.2. Stromal APC Signaling

Apart from genetic alterations in tumor cells, mutations in the stromal component also promote the progression of benign endometrial polyps to an advanced metastatic stage. Our study has shown the crucial role of stromal adenomatous polyposis coli (APC) in controlling the proliferative potential of the endometrial epithelium [47]. APC is a multi-domain protein that regulates Wnt signaling by controlling the availability of β-catenin. In addition, APC interacts with several other proteins to regulate various cellular processes including cell proliferation, differentiation, and migration. However, stromal deletion of APC contributes to the development of EC. Histologic analyses of an *APC^cKO^* mutant mouse model has shown the progressive development of endometrial hyperplasia, increase in stromal myofibroblast population, decrease in expression of estrogen receptor α (ERα), progesterone receptor (PR), and higher levels of VEGF and SDF-1, which collectively indicates an advanced stage of EC [47].

#### 2.3.3. Stromal LKB1 Signaling

LKB1 (Liver Kinase B1) is a negative regulator of the mTOR pathway. Loss of stromal LKB1 signaling plays a major role in EC progression [48]. The stromal cell-specific loss of *Lkb1* induces high-grade EC in the uterine epithelium by activating the mammalian target of rapamycin complex 1 (mTORC1) [48]. LKB1 inactivation also results in an abnormal cell autonomous production of the inflammatory cytokine-chemokine (C-C motif) ligand 2 (CCL2) which facilitates the recruitment of macrophages to promote tumor growth [49].

#### 2.3.4. Stromal HAND2 Signaling 

Hypermethylation of the *HAND2* (Heart And Neural crest Derivatives expressed 2) gene in the endometrial stroma significantly contributes to the development of EC. Epigenome-wide analysis of human EC patients’ tissue samples shows hypermethylation of the *HAND2* gene in the endometrial stroma [50]. Interestingly, a transgenic mouse model harboring *HAND2* knockout has been shown to develop precancerous endometrial lesions [50]. 

#### 2.3.5. Stromal VEGF Signaling

The majority of EC cells express epithelial membrane protein-2 (EMP2) on their cell surface. EMP2 is a novel oncogene which promotes tumor angiogenesis and endothelial cell tube formation through increased secretion of vascular endothelial growth factor (VEGF) [51]. EMP2 activates hypoxia-inducible factor 1-alpha (HIF-1α) in a hypoxic microenvironment through the FAK (Focal Adhesion Kinase)-Src signaling axis and upregulates VEGF expression [51], see Figure 2g. Upregulated VEGF in stroma binds to the VEGF receptor (VEGFR) on tumor cells to stimulate growth and proliferation. Moreover, an increased level of VEGF expression in patients with endometrioid EC is a predictor of poor prognosis [52]. 

#### 2.3.6. Stromal Estrogen Signaling

Steroid signals in the stroma also contribute to EC progression. Stromal estrogen receptor (ERα) mediates the mitogenic effects of estrogen on endometrial cell proliferation [34]. The existing evidence clearly demonstrates the contribution of unopposed estrogen towards tumorigenesis and progression of endometrial carcinoma [53]. In postmenopausal women, despite low levels of circulating plasma estrogen, the crosstalk of tumor and stromal cells contribute to an increase in aromatase activity and estrogen biosynthesis [35]. The positive feedback loop between IL-6, aromatase, and in situ estrogen maintains elevated estrogen signaling in the EC microenvironment [36]. In situ, estrogen binds to ERα and induces the upregulation of IL-6 in the EC cell via activation of the NF-κB (Nuclear Factor kappa-light-chain-enhancer of activated B cells) pathway [37]. IL-6 further stimulates aromatase expression in the endometrial stromal cell through the IL-6 receptor. Increased aromatase expression leads to the synthesis of more estrogen, which causes endometrial hyperplasia and cancer [36], see Figure 2h. 

### 2.4. Paracrine Effects of Adipocytes in the EC Microenvironment

Adipocytes are the predominant cell type in adipose tissue, which maintains the energy homeostasis of the body [38]. In obese individuals, the hypertrophied adipocytes secrete important amounts of adipokines and growth factors which provide an energy source for the tumor cells to grow and invade [54]. Increased adiposity or obesity is not only a major risk factor for cardiovascular disease and type-2 diabetes but also an important cause for multiple types of cancers including EC [55,56]. Approximately 57% of EC cases in the United States are related to obesity, which supports the notion that obesity is a major risk factor for EC [57]. In fact, high BMI (Body Mass Index) is strongly associated with the development of EC [58]. In a recent meta-analysis study, Renehan et al. show that each increase in BMI of 5 kg/m^2^ significantly increases a woman’s risk of developing EC with a relative risk of 1.59 [59]. Epidemiologic studies also revealed that the risk of EC is higher in western countries as well as in women who live a sedentary lifestyle [60,61]. 

The mechanism by which obesity promotes tumorigenesis varies by cancer site. In obese women, the paracrine signaling from visceral adipocytes in the vicinity of the uterus (fat depots in the omentum and bowel mesentery) elevates EC cell proliferation. In the case of EC, the potential players involved in the interaction of adipocytes and EC cells are elevated estrogen levels, insulin, insulin growth factor-1 (IGF-1), adipokines (leptin, resistin), cytokines (IL-6, TNFα), and VEGF-mTOR signaling [39,56,62,63].

### 2.5. Mechanism Relating Obesity or Adiposity to EC Risk

#### 2.5.1. Leptin Resistance

**Figure 3:**
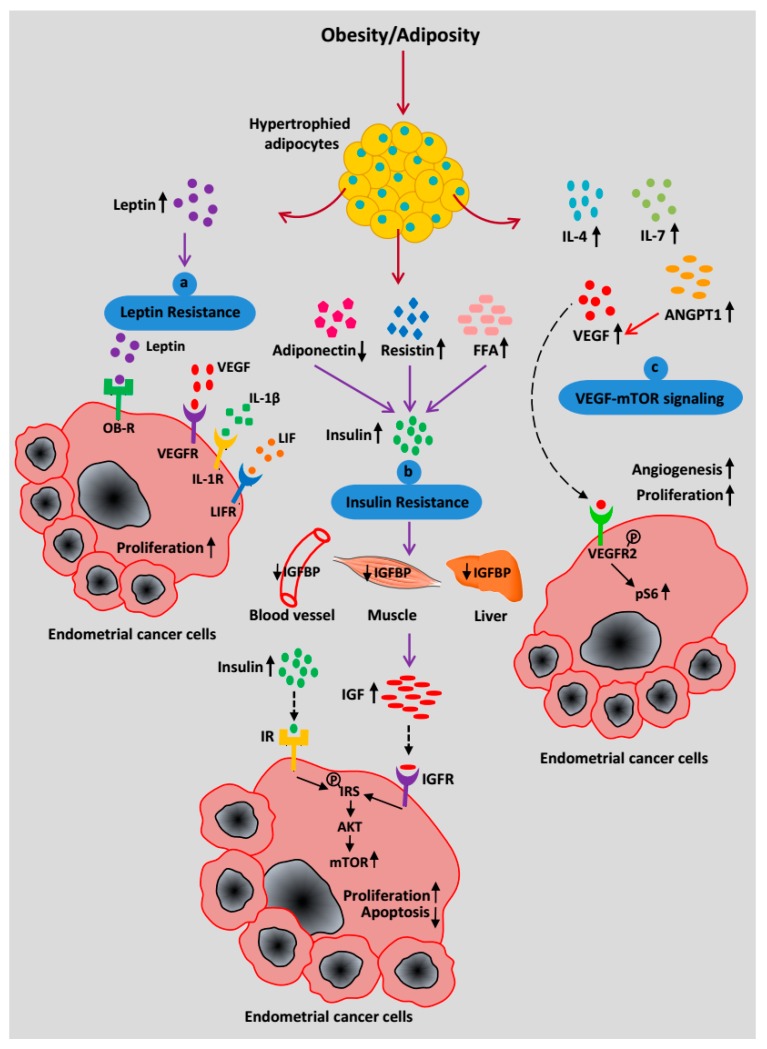
Systemic effects of increased adiposity on EC progression. (**a**) A high level of circulating leptin binds to its receptor OB-R to trigger a signaling pathway as well as recruit pro-angiogenic factors such as VEGF, IL-1β, and LIF to induce cancer progression and metastasis. (**b**) Increased free fatty acids (FFA), resistin, and decreased adiponectin secretion contribute to insulin resistance, which leads to an increase in insulin synthesis. Hyperinsulinemia is associated with decreased bioavailability of IGFBP and the simultaneous increase in IGF-1 production. Insulin and IGF-1 signal through IR and IGFR respectively to promote EC progression via mTOR activation. (**c**) Hypertrophied adipocytes secrete an increasing amount of pro-inflammatory cytokines (IL-4, IL-7), ANGPT1, and VEGF to infiltrate endothelial cells, which facilitates angiogenesis. VEGF acts as a key mediator of the EC cell-adipocyte interaction and binds to its receptor, VEGFR2, on the EC cell surface. Phosphorylation of VEGFR2 activates downstream targets and upregulates the mTOR pathway through a high pS6 level.

Leptin, a pleiotropic cytokine, has a significant contribution to EC progression [40]. Leptin is a small non-glycosylated protein coded by obese (*OB*) gene and secreted by adipocytes. As a primary function, it regulates energy intake and expenditure. Upon leptin resistance, obese individuals exhibit higher levels of circulating leptin [64]. Leptin signals through binding to its receptor (OB-R) and triggers several canonical and non-canonical signaling pathways [65]. Reported studies have shown overexpression of OB-R in EC cells compared to normal endometrial cells [40,41]. In EC cells, leptin signaling is also associated with the recruitment of several pro-angiogenic factors such as VEGF, IL-1β, LIF (Leukemia Inhibitory Factor) to their respective receptors, VEGFR, IL-1R, and LIF receptor (LIFR) [40], see Figure 3a. These signals collectively contribute to endometrial carcinogenesis.

#### 2.5.2. Insulin Resistance

In obesity, due to excess visceral adiposity, the level of circulating free fatty acids (FFA) increases along with peptide hormones such as leptin, resistin, and TNFα while the level of adiponectin decreases. The altered secretion of adipokines leads to insulin resistance (reduced metabolic response of muscle, liver, and adipose tissues to insulin). Insulin resistance results in hyperinsulinemia, which reduces the levels of IGF-1 binding proteins (IGFBP1, IGFBP2) and thereby increases IGF-1 availability [42]. Increased levels of bioavailable insulin and IGF-1 signal through the insulin receptor (IR) and IGF-1 receptor (IGF-1R), respectively, to promote EC cell proliferation [66]. Ligand binding to IR and IGF-1R phosphorylates insulin receptor substrate 1 (IRS-1), which further results in activation of the PI3K/AKT/mTOR pathway and promotes EC cell survival and proliferation [67], see Figure 3b.

#### 2.5.3. EC Cell-Adipocyte Interactions

In high BMI patients, the hypertrophied adipocytes secrete increasing amounts of pro-inflammatory cytokines such as MCP-1, TNFα, IL-6, and IL-8 [68]. The increased level of inflammatory cytokines results in the infiltration of lymphocytes, macrophages, and endothelial cells, which alters the adipose tissue microenvironment. In a paracrine manner, these secreted cytokines also promote the proliferation of EC cells [54]. Moreover, adipocytes in contact with cancer cells differentiate and reprogramme into cancer-associated adipocytes (CAA) [69]. CAA secretes adipokines to simulate adhesion, migration, and invasion of tumor cells.

#### 2.5.4. Adipose-Derived VEGF-mTOR Signaling

In addition to the secretion of cytokines and adipokines, our recent findings suggest that in obese individuals, visceral adipose tissue (VAT) secrete a surplus of VEGF. Using EC tissue biopsies and an obese mouse model, our results ascertain that high VEGF in visceral adipocytes promotes vasculature in the uterus and upregulates mTOR signaling in the endometrial glands, see Figure 3c [63]. Thus, in a paracrine manner, the hypertrophied adipocytes in obese women stimulate endometrial hyperplasia and/or cancer through the VEGF-mTOR signaling axis [63].

#### 2.5.5. Adipocyte-Derived Estrogen Signaling

Obesity or adiposity influences the synthesis of endogenous sex steroids, such as estrogens in postmenopausal women [54]. In adipocytes, 17β-hydroxysteroid dehydrogenase converts androstenedione to testosterone and estrone to estradiol [54]. Moreover, adipose tissue is a predominant source of the enzyme aromatase which converts androstenedione to estrone and testosterone to estradiol [43]. Thus, obese individuals have high circulating levels of estrone and estradiol, which leads to excess estrogen production. Obesity also leads to hyperinsulinemia and increases IGF1 bioactivity, which, in turn, results in the reduced hepatic synthesis of sex hormone binding globulin (SHBG) [70]. SHBG has a high binding affinity for testosterone and estradiol and maintains a normal hormone level. Whereas, the adiposity-induced decrease in SHBG leads to an increase in bioavailable estradiol and, subsequently, an elevated estrogen level [70]. Endometrial cells express estrogen receptor (ER) and are sensitive to estrogen stimulus, which induces endometrial hyperplasia [71]. Thus, in obesity, the phenomenon of estrogen generation by adipocytes is an important risk factor for EC development.

## 3. Targeting the EC Microenvironment for Chemoprevention

Like in most cancers, genetic mutations in oncogenes and/or tumor suppressor genes result in deregulated cell division in the endometrium, which leads to the development of EC. Current targeted approaches aim to eliminate tumor cells by disrupting the activated cancer-signaling pathway such as PI3K/AKT/mTOR signaling which is well-known to be upregulated in EC [72,73]. Although most tumor cells show a good initial response to chemotherapy, EC cells eventually develop chemoresistance and disease relapse. Most of the targeted therapies in EC are used against a single dominant driver mutation or to block essential biochemical pathways and mutant proteins that are required for tumor cell growth and survival. However, most EC patients exhibit genetic heterogeneity [74], which leads to a limited therapeutic response of targeted agents. Moreover, the complex and heterogeneous TME mediates resistance of the solid tumor to drugs. Therefore, instead of directly targeting tumor cells, diminution of growth factors which activate the cancer-promoting signaling pathway might be more promising. Increasing evidence suggests that disruption of the TME that facilitates tumor cell infiltration may provide an additional level of therapeutic intervention as well as serve as a novel paradigm to treat cancers [12,75]. In this context, as discussed previously, myofibroblast-secreted HGF activates AKT and promotes endometrial cell proliferation [22], which can be controlled using inhibitors that may reduce the excess synthesis of HGF by myofibroblasts. Similarly, adipocyte-secreted VEGF also stimulates the mTOR pathway in the uterus [63], which may be suppressed by controlling the excess VEGF secretion via the use of inhibitors. In addition, modulation of progesterone receptor signaling in the EC microenvironment by progesterone therapy results in resolution of endometrial tumor cells [76,77,78,79]. Investigations also show the effectiveness of immunotherapy such as therapeutic cancer vaccines against EC [80,81,82]. Moreover, as already described in this review, inhibition of the ECM-derived TGF-β signaling by small molecule inhibitors significantly suppresses EC metastasis beyond the uterus [46]. Given the cytotoxic effect of chemotherapy, despite killing tumor cells, perturbation of microenvironment-derived signals may provide a broad roadmap to convert these challenges into opportunities. This strategy may render the idea of chemoprevention (such as hormonal therapy, immunotherapy) and may decrease the side effects of chemo drugs on other cell types. Thus, targeting the stromal component of the TME can more effectively demolish tumor cells in EC patients and improve quality of life. 

## 4. Conclusions

High-grade or metastatic EC has long been associated with substantial changes in the extracellular microenvironment. Moreover, it is increasingly clear that a single insult (genetic mutations) is not sufficient to initiate the disease, and that a second hit (microenvironment-derived signals) may be required to drive tumor progression. The neoplastic and non-neoplastic cells in the microenvironment communicate in concert to produce a stromal microenvironment that is conducive to endometrial carcinogenesis. Although it is well demonstrated that the TME can foster a pro-tumor milieu, the precise mechanism by which tumor and stromal cells communicate for the formation of a favorable environment remains elusive. Interestingly, recent evidence in other cancers has shown that nerves present in the TME also promote tumor progression and that the nerve-cancer cell crosstalk is essential for cancer growth and metastasis [83,84]. Whether the neural compartment is involved in EC should be investigated, and opens a new perspective for a better understanding of the multi-parametric nature of TME in EC. Thereby, further in vivo and clinical study of the therapeutic targeting of EC microenvironment is warranted. Looking forward, we believe that this rapidly moving field will guide the rational design of combinational therapies to target both the EC cell and its microenvironment.

## Figures and Tables

**Table 1:** The stromal cell population in endometrial cancer (EC) microenvironment has distinct functions during tumorigenesis.

Cell Type	Roles in Endometrial Cancer		References
	Anti-Tumorigenic	Pro-Tumorigenic	
Fibroblasts	Release growth factors and maintain tissue integrity	Limited	[17,18]
Myofibroblasts	Facilitate deposition of collagen fibers in ECM and involve in wound healing	Chronic secretion of HGF and CXCL12 promote EC cell proliferation and angiogenesis	[19,20,21,22,23,24,25]
Cancer-associated fibroblasts (CAF)	Limited	ECM remodeling Provide oncogenic signals and secrete cytokines for infiltration of tumor cells and macrophages	[26,27,28]
Macrophages (M1)	Provide pro-inflammatory response and secrete TH1 cytokines	Limited	[29]
Tumor-associated macrophages (M2)	Limited	Provide anti-inflammatory response and secrete TH2 cytokines Support angiogenesis and invasion	[30,31,32,33]
Uterine stroma	Provides structural support to endometrium	Expression of aromatase synthesizes in situ E2 to induce endometrial hyperplasia	[34,35,36,37]
Adipocytes	Function as an endocrine organ, accumulate lipids and store as energy	Limited	[38]
Cancer-associated adipocytes (CAA)	Limited	Chronic adipokine and cytokine secretion leads to leptin and insulin resistance Aromatase synthesis results in excess estrogen production	[39,40,41,42,43]
